# Increasing the complexity of isolated musical chords benefits concurrent associative memory formation

**DOI:** 10.1038/s41598-023-34345-y

**Published:** 2023-05-09

**Authors:** Nawras Kurzom, Ilaria Lorenzi, Avi Mendelsohn

**Affiliations:** 1 Sagol Department of Neurobiology, University of Haifa, Haifa, Israel; 2 The Institute of Information Processing and Decision Making (IIPDM), University of Haifa, Haifa, Israel; 3 Department of Biology, University of Pisa, Pisa, Italy

**Keywords:** Psychology, Human behaviour, Learning and memory

## Abstract

The effects of background music on learning and memory are inconsistent, partially due to the intrinsic complexity and diversity of music, as well as variability in music perception and preference. By stripping down musical harmony to its building blocks, namely discrete chords, we explored their effects on memory formation of unfamiliar word-image associations. Chords, defined as two or more simultaneously played notes, differ in the number of tones and inter-tone intervals, yielding varying degrees of harmonic complexity, which translate into a continuum of consonance to dissonance percepts. In the current study, participants heard four different types of musical chords (major, minor, medium complex, and high complex chords) while they learned new word-image pairs of a foreign language. One day later, their memory for the word-image pairs was tested, along with a chord rating session, in which they were required to assess the musical chords in terms of perceived valence, tension, and the extent to which the chords grabbed their attention. We found that musical chords containing dissonant elements were associated with higher memory performance for the word-image pairs compared with consonant chords. Moreover, tension positively mediated the relationship between roughness (a key feature of complexity) and memory, while valence negatively mediated this relationship. The reported findings are discussed in light of the effects that basic musical features have on tension and attention, in turn affecting cognitive processes of associative learning.

## Introduction

Music is a unique stimulus that exerts various emotional responses^1–3^ and can thus influence concurrent cognitive processing^4,5^. The apparent effects of music on human emotion have been observed throughout history and cultures, yet the effects that various musical properties have on cognitive functions remain inconclusive despite its being a long-standing question^6,7^. This inconsistency might partially be explained by the complexity and diverse types of music itself^8,9^, the variety of cognitive tasks^10^, and the individual differences and musical preferences of the learners^5^. A unique property of music is that it has the power to directly produce a wide range of emotions, despite its abstract nature^11,12^. Indeed, music activates brain regions associated with emotional processing^3^ and can modulate autonomic and peripheral reactions, such as galvanic skin responses^1,13,14^, pupil size^15,16^, and heart rate^1,17^. According to one approach, music that is subjectively experienced as ‘pleasant’ might foster learning by generating an emotional response that, in turn, enhances perceptual and cognitive processing^18,19^. Other approaches account for the cognitive limitations that background music might impose on concurrent cognitive-demanding tasks. In these cases, the learner’s cognitive load might be overtaxed by the presence of two concurrent stimuli, limiting cognitive resources (see, for instance, the cognitive capacity model^19^).

In the current study, we investigated the effects of music on cognition by employing musical elements with a reduced set of variables, namely discrete chords with varying degrees of complexity. Chords are the building blocks of ‘Western’ harmony^20^, which enable the construction of chord progressions that constitute musical harmony. Each chord is composed of two or more tones with specific pitch intervals, yielding common types of musical chords, such as major, minor, diminished, augmented, etc. Musical chords range in their consonance and dissonance degrees, though the definitions of these terms are a matter of debate^21^. By and large, consonance is considered as sounds that please the ear, whereas dissonance is sensed as less harmonious and at times discomforting. Some experiments have argued that musical harmony is based on inborn mechanisms^12^, as consonance is preferred over dissonance by human newborns^22^ and even by infant Chimpanzees^23^. As for adults, previous studies on diverse populations show consistent results highlighting the accurate perception of different types of chords (by both musicians and non-musicians), such that major chords are perceived as more consonant than minor chords, which are in turn perceived as more consonant than diminished and augmented chords^24^. This trend was observed in various populations, including American musicians, American non-musicians, and Japanese non-musicians (see for example^24,25^).

Recent studies attempting to model consonance and dissonance perception have demonstrated that the perception of consonance can be primarily explained by degrees of roughness, harmonicity, and familiarity^26^. Of specific significance to this study is the dimension of roughness, which relates to the beating of several frequencies, such that close frequencies interact to produce interference, which may be perceived as dissonant^27^. The use of roughness in this study was favored as it is a well-established indicator of musical emotions^28^, in addition to its strength and reliability in predicting dissonance compared to other features, such as harmonicity^21,27^. Interestingly, the above-mentioned classification of consonance/dissonance does not necessarily correspond to the subjective *preference* of chords. For instance, Lahdelma and Eerola^33,34^ found that listeners favor mildly dissonant chords over maximally consonant and dissonant ones. It has been suggested that the interaction between complexity and perceived likability/preference takes an Inverted-U shape. This idea dates back to Fechner’s principle of avoidance of extremes (1876), which argues that aesthetic pleasure is derived from moderate complexity, diverting from over-simplicity on the one extreme and from chaos on the other^29^. Nonetheless, the effects of musical complexity on declarative memory are inconclusive as of yet^6^.

In the current study, chords were selected to vary in their complexity, operationalized here as degrees of roughness. Additionally, each chord was subjectively evaluated by participants in terms of valence (ranging from pleasing to aversive), felt-tension (ranging from calm to tense), and attention-grabbing (ranging from low to high). Arousal and valence are considered two crucial dimensions of emotions, and our understanding of how emotions affect memory relies on the effects of these dimensions^30^. Previous research highlights the existence of two sub-dimensions of arousal: energetic arousal, encompassing experiences ranging from energetic states to exhaustion, and tension arousal, consisting of states ranging from tension anxiety to calmness and quietness^31,32^. In the context of chord perception, previous studies have distinguished between dimensions related to energy and tension, demonstrating positive correlations between these two elements^33,34^. Given the overlap between subjective ratings of tension and energy, we opted to focus solely on tension. Nevertheless, we measured implicit responses of physical arousal by tracking pupil size changes to the utilized chords. Moreover, the degree to which musical chords appear to grab attention is also imperative in this context, as some musical chords may utilize more attentional resources, which could in turn affect learning outcomes^35–37^.

We aimed at examining how the perception and roughness of different types of musical chords may affect the formation of (unknown) word-image paired associates. Such paired-associate learning is contingent upon neural substrates involved in the binding of discrete information units, which can be explicitly retrieved at a later time^36,38^. In order to achieve this aim, participants were invited to participate in a 2-day experiment, where they learned new German word-image pairs accompanied by different types of musical chords (varying in their complexity) or devoid of musical input (silence). On the second day, participants were asked to determine the correct image associated with each word, and to provide subjective assessments with regards to the musical chords. An additional eye-tracking experiment was conducted to measure pupil dilation changes during the passive listening to the same chords as in the main experiment. Our findings demonstrate that regardless of musical background, individuals successfully perceive variations in chord features corresponding to pleasantness, tension evoking and attention grabbing. A relationship was found between chord type and memory performance, such that major chords were detrimental to learning, whereas chord complexity increased subsequent memory performance. In addition, valence and tension were found to mediate the relationship between the roughness of chords and overall memory performance, indicating that acoustic elements corresponding to the consonance-dissonance continuum are perceived differently, and in turn, affect memory for concurrent associations.

## Methods

### Participants

Fifty participants (17 males, mean age = 27.23, SD 5.62; 33 females, mean age = 26, SD 6.23) took part in the memory experiment, and an additional 42 participants completed the eye-tracking experiment (15 males, mean age = 28.4, SD 6.36, 27 females, mean age = 28.3, SD 4.9). A power analysis (calculated using GPower software^39^) indicated that 35 participants were required for detecting repeated-measures ANOVA effects with a statistical power at a 0.95 level and an alpha of 0.05. Therefore, our samples of 50 (in the main memory experiment) and 42 (in the eye-tracking experiment) were adequately powered to detect a medium effect size (Cohen’s f = 0.4). None of the participants were professional musicians, though 11 participants from the memory experiment played a musical instrument, and 17 of the eye-tracking experiment (see Table 1). Additionally, none of the participants were native German speakers and their native languages were diverse, including Hebrew, Arabic, Mandarin Chinese, and Hindi. The inclusion criteria for participating in the study were: (1) complete ignorance of the German language, (2) no reported auditory problems or acoustical sensitivity, and (3) the absence of an ADHD or other attention-related diagnosis. Participants remained naïve as to the aims and purposes of the study. Outliers (participants with overall memory performance below two standard deviations from the mean) were excluded from the memory data set (4 in total), resulting in 46 valid cases for analysis. The experiment was approved by the ethics committee of the Psychology Department of the University of Haifa, and participants were remunerated for their participation.

**Table 1:** Post-experiment responses to the musical background questionnaire for the memory and eye-tracking experiments.

	Memory experiment	Eye-tracking experiment
Overall interest in music (scale 1–5) ± SE	4.03 ± 0.14	4.04 ± 0.15
Overall musical ability (scale 1–5) ± SE	2.32 ± 0.18	2.90 ± 0.18
Hours listening to music (per week) ± SE	12.31 ± 1.62	11.95 ± 1.37
Engagement in music (scale 1–5) ± SE	3.06 ± 0.17	2.66 ± 0.17
Playing a musical instrument	No musical instrument (n = 35) Playing a musical instrument (n = 11)	No musical instrument (n = 25) Playing a musical instrument (n = 17)
Exposure to classical music	Listening often to classical music (n = 22) Not listening to classical music (n = 24)	Listening often to classical music (n = 20) Not listening to classical music (n = 22)

### Auditory stimuli

The auditory stimuli consisted of 80 musical chords selected from a database of chords previously recorded and made publicly available along with their acoustical measurements from a study by Lahdelma and Eerola^33^. These chords were divided into four categories of major chords, minor chords, Medium complex chords, and highly complex chords (press on each chord to listen to an example chord from each category), with 20 chords in each category, randomized in the pitch of the chords’ root (see Table 2).

**Table 2:** Types of chords used in the memory experiment and the rating sessions.

	Major chords	Minor chords	Medium complex chords	Highly complex chords
Nr. of tones	3	3	4	5/6
Chord types	Normal 3-tone major triads	Normal 3-tone minor triads	Sixth and m7 chords (tetrads)	Hexatonic, Dom7plus11, maj9, Neapolitan (pentads and hexads)
Average roughness	0.22 (SD = 0.07)	0.18 (SD = 0.06)	0.65 (SD = 0.69)	2.04 (SD = 0.66)

Roughness data were collected based on measurements by Lahdelma and Eerola^33,34^. For the complete set and description of chords, see Supplementary Table S1 in Supplementary Material.

The criteria for classifying the chord complexity here depend on the number of tones and the degree of roughness of each given chord. Acoustic roughness is related to the degree of perceived dissonance, brought about by physical interactions of sound waves with similar frequencies^40^. The term roughness may be preferred over sensory dissonance, as the former applies to different types of sound beyond music^41^. Analysis of roughness degrees revealed a significant difference in roughness among the different chord types (F_3,76_ = 65.39, P < 0.001), such that highly complex chords had the highest roughness. Bonferroni corrected post-hoc comparisons indicated that roughness scores for medium complex chords were significantly higher than those of major chords (P < 0.05) and minor chords (P < 0.05), while roughness scores for highly complex chords were significantly higher than those of major chords (P < 0.001), minor chords (P < 0.001), and medium complex chords (P < 0.001). In the pool of chords utilized in this study, major and minor chords had the same number of tones, and their roughness values were not statistically different; nevertheless, the major-minor distinction stems from well-established differences associated with the emotional response they evoke^33,42^.

### Visual and verbal stimuli

**Figure 1:**
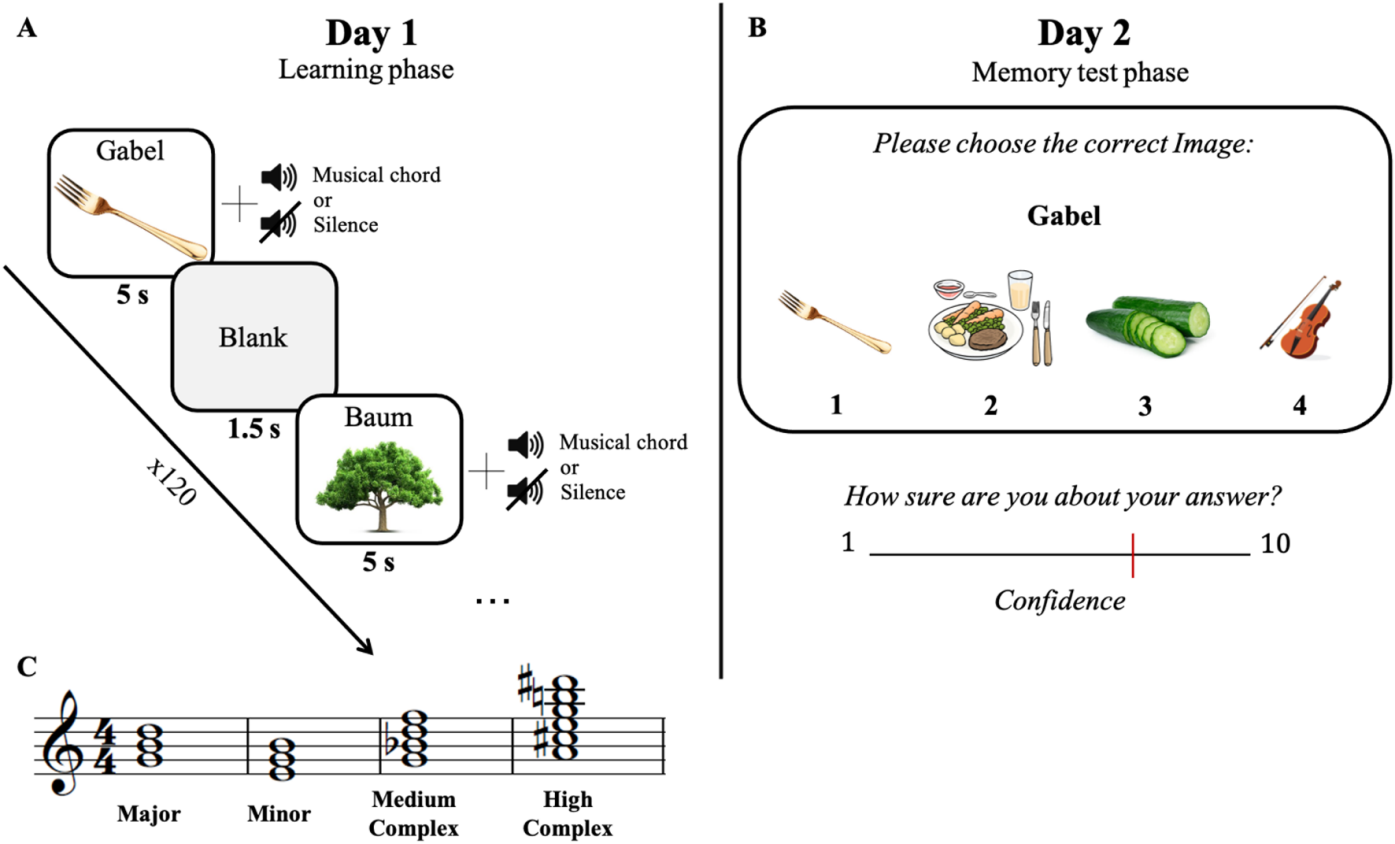
Schematic illustration of the experimental design. (**A**) Learning phase. Schematic timeline and examples of two chord-image-word stimuli presented during learning. (**B**) In the memory test phase, carried out one day after learning, participants were required to associate each presented word with its corresponding image, and to rate their confidence regarding the answer. (**C**) Musical notations of selected musical chords from each of the chord categories used in the study.

One hundred and twenty emotionally-neutral German words and 120 corresponding images were selected from open-access databases: The verbal stimuli were obtained from the database of noun associations in German^43^, and visual stimuli were collected from the Bank of Standardized Stimuli^44^. Semantic relationships between these words and words in other languages were avoided as much as possible. To examine the memorability of the words regardless of musical manipulation, a post-experiment analysis was conducted by calculating the group distribution of memory performance for each word. Regardless of musical chord category, memory for the word-images pairs was normally distributed (mean % of correct answers = 73.67%, mode = 72%, range = 57.5–92.5%, see Supplementary Fig. S1). In the learning phase, the stimuli were presented as word-image pairs along with a single chord/silence. In the memory phase, each word was presented along with four images, from which participants were required to choose the image they thought corresponded to the presented word (see Fig. 1).

### Main experimental procedure

The main experiment consisted of two major phases: a learning phase and a memory test phase (24 h later). During the learning phase, participants were presented with discrete chord-word-picture events, each consisting of a written German word (unfamiliar to participants), and an accompanying picture that depicts the word’s meaning, and a randomly assigned chord from one of the above-mentioned chord categories. The experiment included 120 events, 80% of which were accompanied with a chord of a certain type, and 20% devoid of musical stimuli (silence condition). Each event lasted 5 s, followed by an inter-stimulus time interval of 1.5 s. The chord assignment to each word-image pair was randomized for each subject, and the order of the accompanying chords was counter-balanced in order to avoid potential effects of the temporal transitions between the chords.

During the memory test phase (24 h later), participants were presented with 100 words from the encoding phase (maintaining the 80/20% ratio of events with and without chords), accompanied by four optional pictures for each word. Participants were instructed to choose the picture they thought corresponded to the presented word, and to rate the confidence in their answer (see Fig. 1B). Following the memory test, participants listened to all chords used in the encoding session, and were asked to rate each one of them on three separate dimensions of valence (from 1-highly aversive, to 5-highly pleasant), tension (from 1-very calm, to 5-very tense), and the degree to which they felt the chords grabbed their attention (from 1-does not grab attention at all, to 5-highly attention grabbing). Finally, participants were asked to answer a musical background questionnaire (based on the musical background questionnaire developed by Ref.^45^.

### Eye-tracking experiment

A separate group of 42 participants were recruited to undergo an eye-tracking experiment to gauge the physiological responses to the different chord types. This was done in order to examine possible physiological responses to different degrees of chord complexity, and to assess the relationship between pupil dilation and subjective ratings of tension, valence, and attention grabbing. This allowed for not only investigation of pupil dilation, but also replication of the chord rating procedure with an independent group of participants. Eight subjects were excluded from further analysis due to technical issues or noisy recordings. Each event began with the presentation of either a white or black circle on screen, followed by a single chord 500 ms later. The circle remained on screen for 3 s. This experiment did not include the word-image stimuli, but only the acoustic stimuli (chords) that were used in the main memory experiment. Using an EyeLink 1000 system (SR Research, Canada), following calibration and validation of the tracked eyes, participants’ eyes were monitored with a sample rate of 500 Hz. For the purpose of this study, pupil diameter was analyzed by calculating for each participant the normalized pupil dilation change (in z-scores) for each chord event for the time period of chord onset to 4 s thereafter. Next, for each participant, the mean pupil response was calculated across the chord events of each category separately, followed by the generation of plots that included the group average pupil dilation responses to each chord category. In the current study, only the responses of the black-circle trials are shown, as these trials instigate increases in pupil dilation. Furthermore, Similar to the memory experiment, participants in this experiment underwent a chords’ rating session followed by a musical background questionnaire after the eye-tracking session.

### Ethical approval and consent to participate

The experiment was approved by the ethics committee of the Psychology Department of the University of Haifa, and procedures were carried out in accordance with relevant guidelines and regulations. All participants provided written informed consent prior to participation.

## Results

### Subjective perception of musical chords

**Figure 2:**
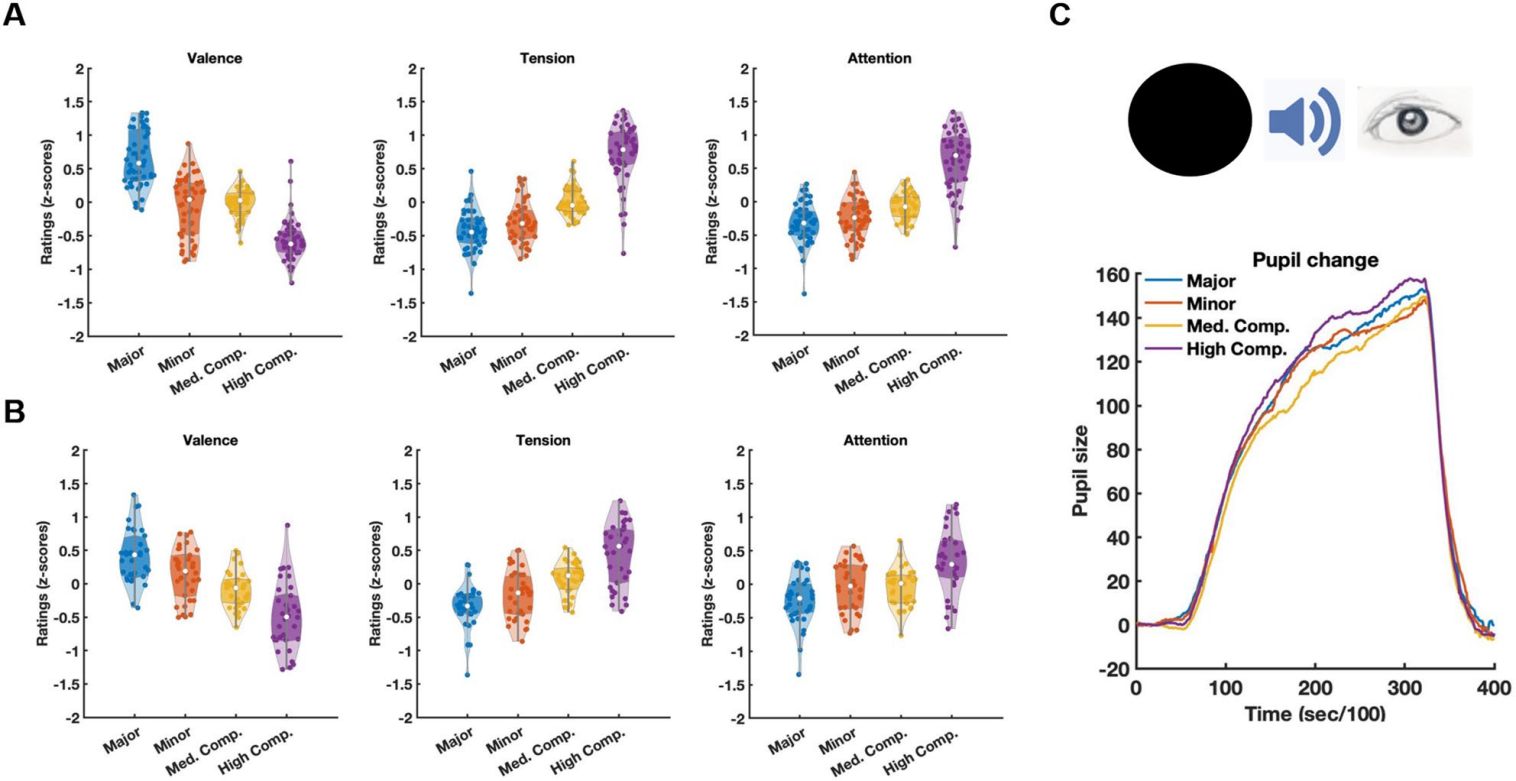
Subjective ratings and pupillometry of chord types. (**A**) Violin plots showing normalized ratings that participants in the main memory experiment provided on indices of valence, tension, and attention-grabbing in response to Major (blue), Minor (orange), Medium complex (yellow), and High complex (purple) chords. Each dot corresponds to a given participant. Significant differences were found among all chord types for valence judgments (F_1,45_ = 225.4, P < 0.0001), tension ratings (F_1,45_ = 132.14, P < 0.0001), and attention-grabbing evaluations (F_1,45_ = 90.5, P < 0.0001). (**B**) Similar violin plots shown for participants of the eye-tracking experiment. Significant differences were found among all chord types for valence judgments (F_1,31_ = 40.4, P < 0.0001), tension (F_1,31_ = 37.8, P < 0.0001), and attention (F_1,32_ = 7.89. P < 0.001). (**C**) Schematic depiction of the experimental layout of an eye-tracking trial, whereby a black circle was presented, followed by the auditory presentation of a single chord. The plot depicts group averages of normalized pupil dilation changes in response to the first five occurrences of each chord type.

Following the memory session, participants were presented with each of the chords used in the experiment and were asked to rate them on a scale from 1 to 5 with respect to three dimensions: valence, tension-evoking, and the degree of attention grabbing. The highest average valence rating was for major chords (mean ± standard error—3.53 ± 0.08), followed by medium complex chords (2.99 ± 0.07), minor chords (2.93 ± 0.11), and finally, highly complex chords (2.46 ± 0.06). Repeated measures ANOVA and post-hoc comparisons between chord-type pairs for all rating results were performed on subject-based normalized (z-score) values. Valence scores differed significantly among chord types (F_1,45_ = 225.4, P < 0.0001), and all chord-type pairs were significantly different (P < 0.0001, Bonferroni corrected) except for minor vs. medium complex chords (see Fig. 2A).

Tension ratings followed the opposite trend, where the highest tension ratings were given for highly complex chords (3.74 ± 0.06), followed by medium complex chords (3.13 ± 0.07), minor chords (2.86 ± 0.09), and major chords (2.75 ± 0.09). Normalized tension ratings differed significantly among chord types (repeated-measures ANOVA—F_1,45_ = 132.14, P < 0.0001), and all chord-type were found to differ significantly (P < 0.001, Bonferroni corrected) except for major vs. minor chords. Similar to tension ratings, the highest average attention-grabbing ratings were assigned to highly complex chords (3.82 ± 0.05), followed by medium complex chords (3.33 ± 0.06), minor chords (3.22 ± 0.07), and finally major chords (3.16 ± 0.07). Normalized attention scores differed significantly among chord-types (F_1,45_ = 90.5, P < 0.0001), and all chord-type pairs differed from one another (P < 0.0001, Bonferroni corrected) except for major vs. minor chords.

Ratings of tension, valence, and the extent to which the chords grabbed attention were also provided by participants in the eye tracking experiment, and the results were strikingly similar to the abovementioned behavioral experiment (Fig. 2B). Valence ratings for major, minor, medium complex, and highly complex chords were 3.55 ± 0.09, 3.21 ± 0.08, 3.06 ± 0.07, and 2.6 ± 0.77, respectively. Repeated-measures ANOVA analysis on normalized valence scores yielded a significant difference among chord-types (F_1,31_ = 40.4, P < 0.0001), and all chord-type pairs differed from one another (P < 0.001, Bonferroni corrected), except for minor vs. major and minor vs. medium complex chords. Average tension ratings for major, minor, medium complex, and highly complex chords were 2.72 ± 0.09, 2.94 ± 0.08, 3.14 ± 0.07, and 3.57 ± 0.09, respectively (F_1,31_ = 37.8, P < 0.0001). All chord-type pairs significantly differed in tension ratings, except for major vs. minor chords (major vs. medium and highly complex chords—P < 0.0001; medium vs. highly complex chords—P < 0.05). Attention-grabbing ratings were 3.29 ± 0.07, 3.45 ± 0.07, 3.38 ± 0.07, and 3.74 ± 0.09, for major, minor, medium, and high complex chords, respectively (F_1,31_ = 14.38, P < 0.001). Here, only high complex chords significantly differed from major chards (P < 0.05).

Pupillometry analysis did not yield significant differences in overall pupil change among the four chord types. However, as shown in Fig. 2C, a trend for enhanced pupil dilation is apparent in the highly complex chords, while the smallest responses are shown for medium complex chords.

### Memory performance by chord type

**Figure 3:**
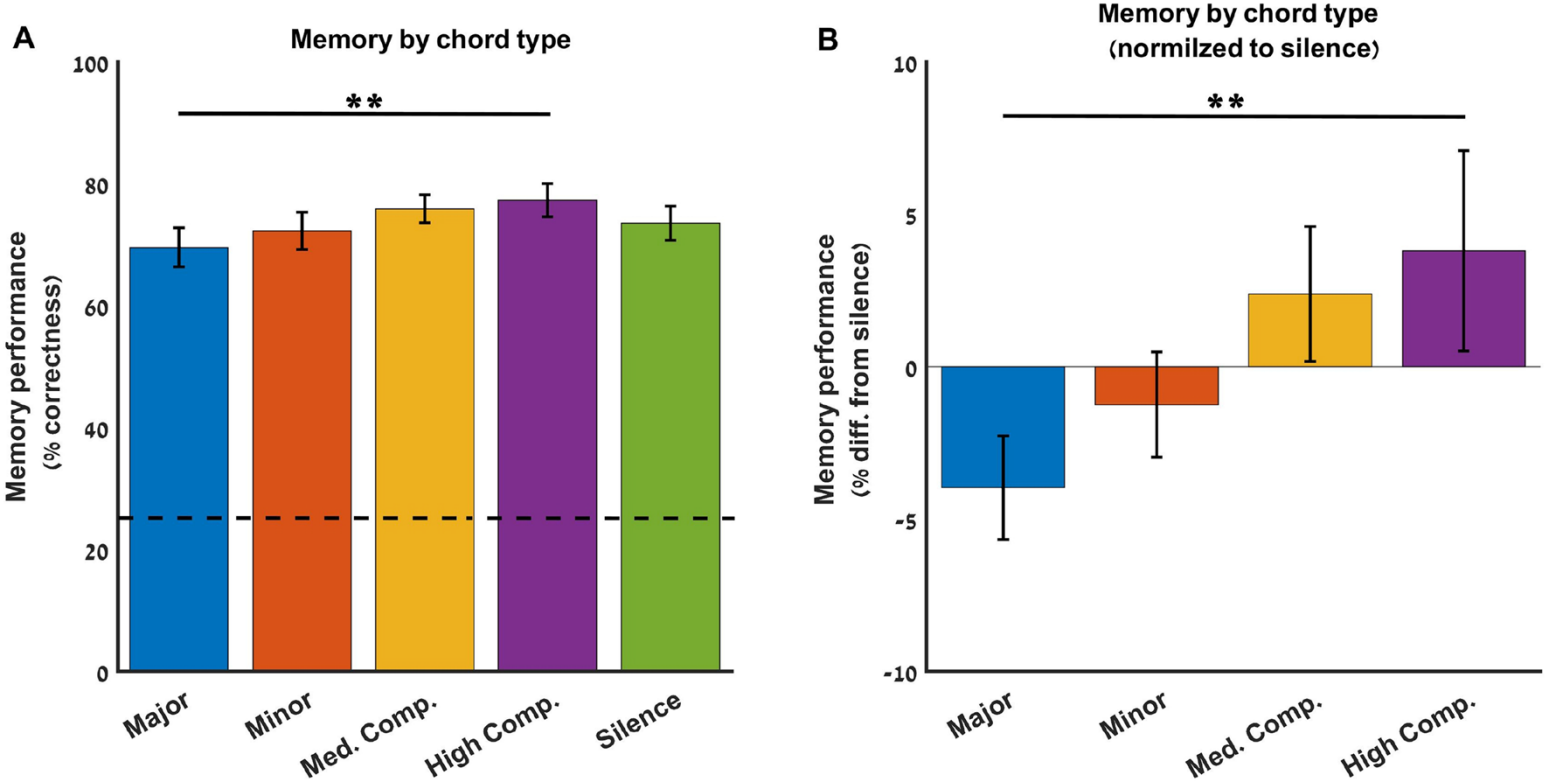
Memory performance by chord type. (**A**) Group mean percentages of correct answers for words learned in the presence of the different chord types and silence. Dashed horizontal line indicates chance level performance. Memory was found to differ among the five conditions (χ^2^_4_ = 10.52, P < 0.05). Post-hoc sign-test contrasts indicated a significant difference between memory performance of the major and high complex chords (P < 0.005, Bonferroni corrected for multiple comparisons). Dashed line indicates chance level memory performance. (**B**) Memory performance (percentage of correct answers) for each chord type after subtraction of memory for words presented in the absence of chords (silence). Memory performance differed among chord types (χ^2^_3_ = 8.24, P < 0.05). Post-hoc sign-test contrasts indicated that the effect stemmed mainly from the significant difference between Major and high complex chords (P < 0.005, Bonferroni corrected for multiple comparisons). Error bars indicate standard error of the mean. *Med.* Medium, *Comp.* Complex.

Figure 3A depicts group means of memory performance, defined for each participant as the percentage of correct images chosen to correspond to the presented words for each chord type. The percentages of correct answers averaged over participants for word-image associations for each chord type were as follows: major chords—69.58 ± 3.18, minor chords—72.29 ± 3.03, medium complex—75.92 ± 2.28, highly complex—77.34 ± 2.75, and silence—73.54 ± 2.79. A non-parametric Friedman test yielded a significant difference in memory performance among the four chord types and silence (χ^2^_4_ = 10.52, P < 0.05), and post-hoc sign-test contrasts indicated a significant difference solely between memory performance of the major vs. high complex chords (P < 0.005, Bonferroni corrected). To account for baseline memory performance regardless of chord type conveyed during learning, we subtracted the memory performance for word-image associations (% of correct answers) of the silence (no chords) condition from each of the other conditions (Fig. 3B). A significant difference in memory performance among chord types was obtained here as well (χ^2^_3_ = 8.24, P < 0.05), stemming from a difference between major and high complex chord conditions (P < 0.05, Bonferroni corrected).

### Interactions between roughness, perception, and memory performance

We next wished to examine the interactions between the acoustic property of chord roughness, chord perception, and memory performance. Therefore, for each single chord, we calculated the percentages of participants who correctly answered the word-image association that was paired with that chord in the learning phase. A grand average was then determined for each chord type, yielding the following averages for major, minor, medium complex and highly complex chords, respectively: 60.97% ± 10.47, 69.72% ± 11.09, 72.33% ± 10.69, and 72.01% ± 10.04. A one-way ANOVA conducted to compare the effect of chord type on the percentages of participants who answered correctly revealed a statistically significant difference (F_3,76_ = 5.05, P < 0.01). Post-hoc t-test comparisons using the Bonferroni corrections indicated that the percentage of participants who successfully formed word-image associations learned in the presence of major chords was significantly lower than for word-image pairs associated with medium complex chords (P < 0.05), and highly complex chords (P < 0.01). Taken together, these results suggest that different types of chords indeed have an effect on memory formation of word-image pairs. Specifically, our results suggest that when participants are presented with major chords while learning new word-image associations, their learning is reduced (compared to the other chord types).

**Figure 4:**
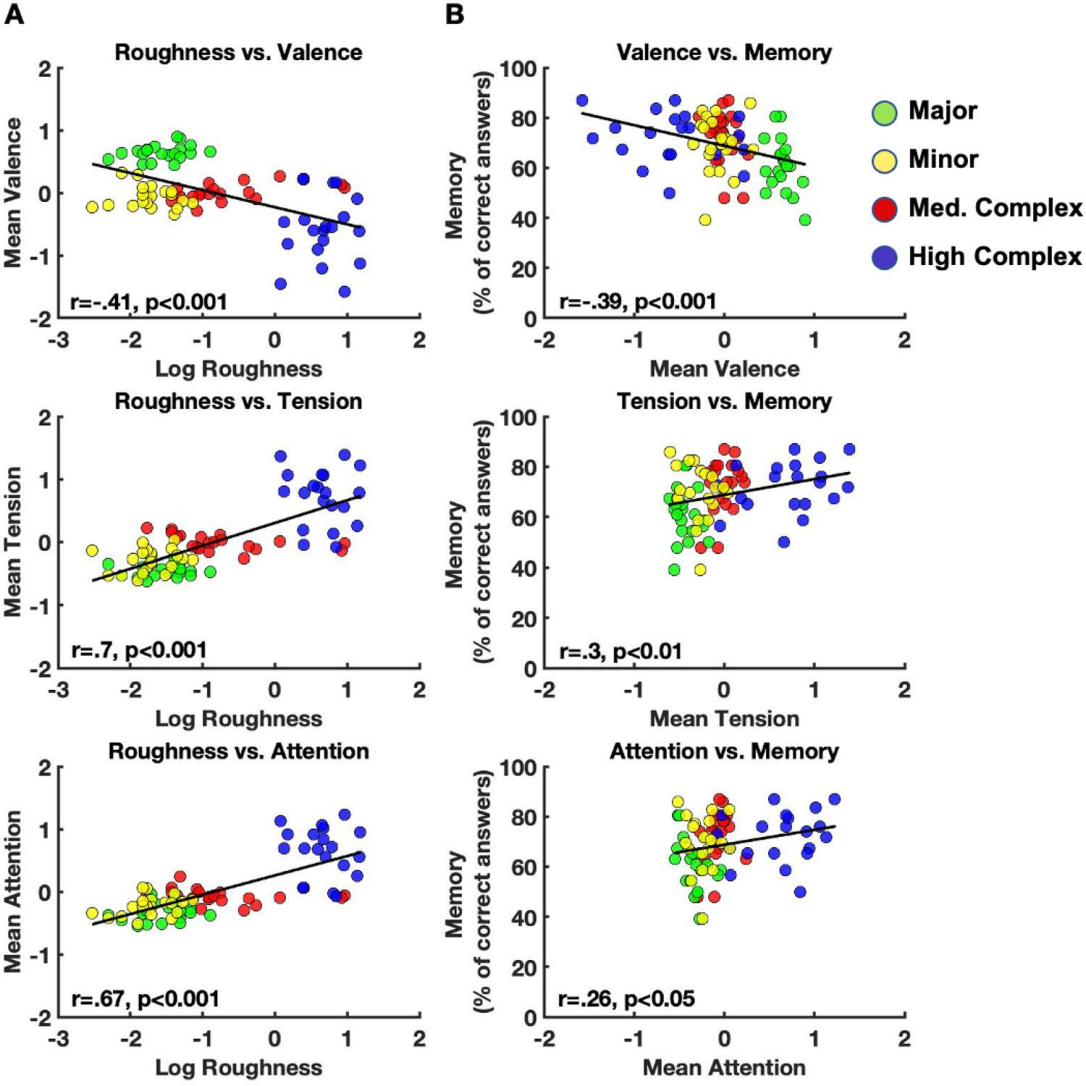
Correlations between roughness vs. ratings and ratings vs. memory performance across all chords. (**A**) Standardized values of ratings scores (y-axis) plotted against the log transform of roughness for each chord (x-axis). (**B**) Percentage of participants who answered correctly for each chord (y-axis) plotted against standardized variables (z-scores) of valence, tension, and attention. Green dots indicate major chords, yellow dots indicate minor chords, red dots indicate medium complex chords, and blue dots indicate highly complex chords. *Med.* Medium.

A similar analysis was performed for each chord separately for ratings of valence, tension, and attention, calculating the average normalized ratings across participants for each chord separately. Similarly, roughness values for each chord were obtained from a previously published article^34^ (see “Methods”). Spearman’s rank correlations were computed to assess the relationship between acoustic roughness and chords’ ratings (valence, tension, attention-grabbing) for each chord type. This analysis yielded a significant negative correlation between valence ratings and roughness (r_44_ = − 0.41, P < 0.001). Moreover, there was a significant positive correlation between tension ratings and roughness (r_44_ = 0.7, P < 0.001), and a significant positive correlation between attention ratings and roughness (r_44_ = 0.67, P < 0.001) (see Fig. 4A). These results imply a relationship between roughness and subjective chord evaluations for all chord types: the higher the roughness of the chord, the more it is perceived as tense or attention-grabbing, and the less it is perceived as pleasant.

We next calculated Spearman’s rank correlations to assess the relationship between subjective chord ratings and the percentage of participants who answered correctly on the memory test across chord types. This analysis demonstrated a statistically significant negative correlation between valence ratings and memory (% of correct participants): r_44_ = − 0.39, P < 0.001, and positive correlations between tension ratings and memory (r_44_ = 0.30, P < 0.01), and attention ratings and memory (r_44_ = 0.26, P < 0.05) (see Fig. 4B).

**Figure 5:**
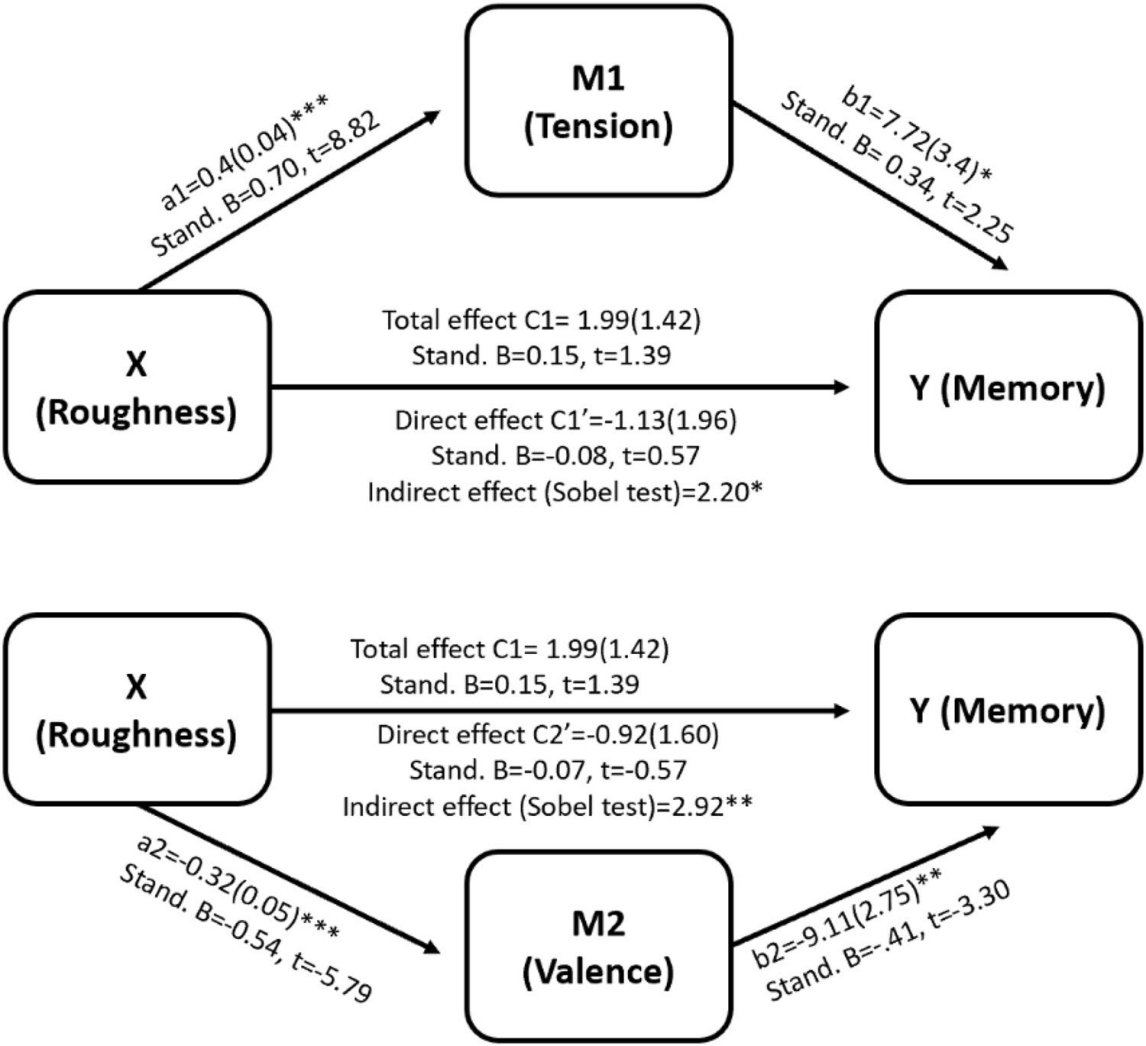
Mediating effects of tension and valence on the relationship between roughness and memory. The non-standardized (a_1_ and a_2_) and standardized values (B) regression coefficients for the association between the independent variable and the mediators are shown for Tension (top) and Valence (bottom). The non-standardized (b_1_ and b_2_) and standardized (B) regression coefficients for the association between the mediators and the dependent variable of memory scores are shown for each mediator as well. Standard errors are depicted in parenthesis, and statistical significance is indicated by asterisks (*P < 0.05, **P < 0.01, ***P < 0.001).

Next, a mediation analysis was performed to assess whether the roughness affects memory performance through the mediation of chord perception ratings. Three Sobel tests were performed independently using valence, tension and attention-grabbing ratings as mediators, with roughness as the independent variable and the percent of participants who answered correctly as the dependent variable. The results of a linear regression showed that roughness was not a significant predictor of memory (no ‘total effect’). Nevertheless, results from the Sobel tests confirmed that the both the perception of tension and valence significantly mediated the relationship between roughness and memory (tension: z = 2.2, P = 0.02; valence: z = 2.92, P = 0.003). Attention was not found to mediate between roughness and memory. In sum, we found significant indirect effects between roughness and memory such that tension positively mediated the effect of roughness on memory performance, and valence negatively mediated the effect. It is worth mentioning that according to Hayes^46^ and Shrout and Bolger^47^, it is appropriate to conclude that there is an indirect mediation effect between X and Y even if the total effect ‘c’ is insignificant. Figure 5 illustrates the opposing mediating effects of tension and valence on the relationship between roughness and memory.

### The role of participants’ musical background

The musical background of participants was assessed using the music background questionnaire developed by Zhao et al.^45^. Averages of the main musical background features are summarized in Table1 (see “Methods”). We did not find statistically significant correlations between the above-mentioned traits and the other main variables in this study (ratings, memory scores, and roughness). However, when each type of chord was assessed independently in relation to the other variables of the study, two statistically significant results were found. First, a Spearman’s rank correlation test conducted on the level of ‘engagement in music’ vs. mean ratings of attention yielded a significant positive correlation (P < 0.01) for all types of chords, except for major chords. This implies that the more the participants engage in music in daily life casual listening, the more they are sensitive to the attention dimension of music chords (except for major chords).

Secondly, the chord ratings of participants who reported that they regularly listened to classical music (n = 22) were compared to those that did not include classical music as one of their music preferences (n = 24). Results showed that those who listened to classical music assigned higher valence to major chords compared to those who did not report listening to classical music (3.8 ± 0.5 and 3.37 ± 0.6, respectively, Wilcoxon test P < 0.05). Similarly, participants who reported that they listen to classical music rated major chords as being less tense (2.4 ± 0.71) compared to those who did not regularly listen to classical music (2.9 ± 0.61, Wilcoxon test, P < 0.05). To summarize, participants’ musical background was not generally correlated with the main study variables, but when examining the role of musical background in relation to subjective ratings of specific chord types, we found a role of the level of music engagement and exposure to classical music on the sensitivity to the perception of major chord.

## Discussion

The aim of this study was to explore the relationship between objective (roughness) and subjective (rating) features of discrete chords on the formation of declarative memory. Overall, participants successfully distinguished between different types of chords in the dimensions of valence, tension, and attention. Our findings indicate that participants formed stronger associations between words and pictures in the presence of chords containing dissonant elements, in comparison to major chords. A mediation analysis indicated that the roughness of chords, defined as the extent of beating frequency components, explained memory strength through a positive effect on tension and a negative effect on valence, which in turn correlated with memory strength.

Music plays a central role in human culture, and is often played in the background of various activities, such as driving, reading, and concentrating on other tasks. Nevertheless, the effects that music exert on learning and memory are diverse and, at times, inconsistent^48^. The reasons for inconclusive conclusions regarding the effects of music on learning include the broad range of musical genres, played in varying tempos and musical instruments, and perceived by individuals with diverse musical preferences^10^. Due to these limitations, we opted to use discrete chords rather than continuous musical pieces to explore their effects on declarative learning. By doing so, we were able to account for particular musical elements related to the consonance-dissonance continuum, as well as perceived pleasantness (valence), and acoustical elements such as sound roughness^49^.

The perception of the chords in terms of valence and tension, as rated here by two separate groups, replicates previous studies showing similar results^24,33,50^. Our findings strengthen the notion that particular pitch intervals are similarly perceived across individuals, whether they possess musical skills or not. Despite the ongoing debate regarding the exact definitions of consonance and dissonant sounds, there is a growing consensus that a key determinant of consonance/dissonance chords is the feature of roughness^26^. Recent studies attempting to model consonance perception have demonstrated that the perception of dissonance can be largely explained by this factor, and to a lesser extent, by the number of notes in a given chord^27^. Our findings support this notion by demonstrating clear relationships between valence and tension and the degree of acoustic roughness of chords. Furthermore, we demonstrate that the acoustic property of roughness influences memory performance through the perception of tension and valence. Notably, the physical roughness of the chords per se did not have a direct effect on memory performance, but rather influenced chord perception and in turn memory performance.

The memory-reducing effect of major chords (during encoding) compared to the more complex chords (containing dissonant characteristics) is in line with previous findings showing that relaxing music significantly reduced memory performance during encoding, while arousing music increased emotionally induced arousal and subsequently enhanced memory strength^51^. Similarly, Bodner et al.^52^ showed that dissonant music had an overall positive effect on participants’ cognitive performance. Nevertheless, the musical stimuli in these studies consisted of continuous music containing other musical elements (rhythm, harmony, tempo, etc.), while in the current study, we utilized distinct isolated music chords devoid of musical context.

The distinction between continuous music and isolated chords is also relevant when considering that the formation of associative memory was superior during the presence of dissonant compared to major/consonant chords. We recently reported that tension perception is negatively correlated with memory performance^13^. However, it is important to note that, unlike our previous work, the current study focuses on isolated single chords without a specific tonal function. When chords are heard in the context of a musical piece, their emotional impact is influenced by their function within the chord progression and the overall musical scale. For example, the presence of a dominant (V) chord in a musical passage may create an expectation for a root (I) chord to follow. In contrast, when chords are isolated from their tonal context, the musical tension is based solely on their acoustic features, such as roughness, rather than any expectations created by their tonal context. This notion resonates with the distinction between tension of expectation and tension of instability for musical chords by Lerdahl^53^. Accordingly, high levels of musical tension combined with expectations could potentially overload cognitive resources and interfere with learning. However, if musical tension is induced solely through the inner properties of the chord (e.g., roughness), it may act as an attentional cue to the main task rather than interfering with it.

Our results point to a trend for increased pupil dilation for highly complex chords as well as higher average ratings of felt tension and attention grabbing (in comparison to major chords). In addition, our results show that tension positively mediated the relationship between the roughness of the chords and memory. These findings imply that chords with dissonant elements may increase feelings of tension, and in turn enhance learning of information present in one’s surroundings. Indeed, mildly arousing stimuli are known to enhance sympathetic responses, in turn strengthening the retention of the encoded information^54^. Pertinent to the current study, Koelsch et al.^50^ have shown that irregular chord sequences (perceived as less pleasant to the ear) evoke stronger blood-oxygenated level-dependent (BOLD) signals in the amygdala, as compared to regular, or pleasing, chord sequences. While the stimuli used in the current study are devoid of musical context (chord progressions or harmonic development), the medium and highly complex chords might be perceived as irregular, compared to the more conventional and pleasing major chords, thus eliciting stronger cognitive performance. It should be noted that what we initially classified (in this experiment) as medium and highly complex chords can both be considered as relatively mildly arousing stimuli. The used musical chords do not contain extremely aversive or fearful elements and do not exert negative real-life implications (compared to other real-life surprising or dangerous stimuli). Moreover, the task used in this study is relatively simple,hence, the addition of the dissonant chords seems to have stimulated some degree of tension, which was in turn translated into increased engagement in the task. Had the task included more aversive stimuli, such as disturbing or loud sounds, we would hypothesize that memory performance would have been negatively affected, in line with the inverted U-shaped relationship between arousal and performance^55^.

One’s musical background can be expected to influence musical perception and concurrent cognitive performance. We did not find such an effect, aside from marginal effects related to the degree of participants’ exposure to classical music on ratings of valence and tension. Our findings indicate that participants with previous exposure to classical music deem major chords as more pleasant and less tense evoking compared to participants who do not regularly listen to classical music. However, the exposure to classical music did not affect their memory performance. Our results are thus largely in line with Lahdelma and Eerola^34^, who found that musical background did not generally affect the perception of the chords’ emotional qualities. Nevertheless, when it comes to memory formation for novel words, some previous studies point out to a possible facilitation effect of musical expertise on second language acquisition (e.g., Refs.^56,57^). However, to the best of our knowledge, none of these studies examined the usage of single musical chords in tandem with a memory test for new word-image pairs.

The current findings may have implications for using auditory stimuli in learning contexts, particularly when learning the vocabulary of a new language. According to our findings, acquiring new information in the presence of music consisting mainly of major chords may be detrimental to memory, while utilizing somewhat irregular musical stimuli that contain dissonant elements as background material for learning could enhance associative memory formation. It should be noted, however, that in actual music, chords are presented within a specific musical context, and the same musical chord can elicit different affective responses depending on the relevant musical context^33^. Future studies could investigate whether the temporal progressions of musical chords within their natural musical context affect the learning processes. Furthermore, future studies are required to examine the role of harmonic complexity on other cognitive tasks, while taking into account the neural underpinnings that underlie the effects of different chord types on memory formation.

To conclude, our results show that memory for word-image associations can be enhanced when studied in the presence of highly complex chords and reduced by the presence of consonant major chords. The effect on memory performance is mediated by a positive relationship with perceived tension, and a negative relationship with perceived pleasantness.

## Supplementary Information

Supplementary Information.

## Data Availability

Musical stimuli used are available in the supplemental information section, and all other data will be available from the corresponding author upon request.
