# Supplementary material for: Increasing the complexity of isolated musical chords benefits concurrent associative memory formation
Source: Sci Rep. 2023 May 9;13:7563. doi: 10.1038/s41598-023-34345-y (PMC10169783; doi:10.1038/s41598-023-34345-y)
Supplement: Supplementary file 1 — Supplementary Information. [file 41598_2023_34345_MOESM1_ESM.pdf]

Supplementary Material

Increasing the complexity of isolated musical chords benefits concurrent associative memory formation

Nawras Kurzom, Ilaria Lorenzi & Avi Mendelsohn

Table S1. Set of all chords used in the encoding session. Chords are categorized by their type, number of tones, and roughness values. All chords were adopted from Lahdelma & Eerola (2016) via the database they made publicly accessible (link below). *Note: numbers of chords mentioned in the first column refer to the original chord numbers used by the authors of the previously-mentioned study.*
https://dataverse.harvard.edu/dataset.xhtml?persistentId=doi:10.7910/DVN/GE5PPL

Nr. Of the chord	Tones	Type	Nr. Of tones	Roughness
MAJOR CHORDS				
1	G-B-D	major	3	0.414926
2	G#-C-D#	major	3	0.23263
3	A-C#-E	major	3	0.271381
4	A#-D-F	major	3	0.307037
5	B-D#-F#	major	3	0.157801
6	C-E-G	major	3	0.305452
7	C#-F-G#	major	3	0.213279
8	D-F#-A	major	3	0.154749
9	D#-G-A#	major	3	0.121839
10	E-G#-B	major	3	0.161477
11	F-A-C	major	3	0.178149
12	B-D-G	major	3	0.185911
13	C-D#-G#	major	3	0.264179
14	C#-E-A	major	3	0.27092
15	D-F-A#	major	3	0.247288
16	D#-F#-B	major	3	0.171229
17	E-G-C	major	3	0.099826
18	F-G#-C#	major	3	0.172348
19	F#-A-D	major	3	0.269088
20	G-A#-D#	major	3	0.248051
MINOR CHORDS				
43	E-G-B	minor	3	0.181361
44	F-G#-C	minor	3	0.245972
45	A#-D-G	minor	3	0.121829
46	B-D#-G#	minor	3	0.217261
47	C-E-A	minor	3	0.172509
48	C#-F-A#	minor	3	0.177628
49	D-F#-B	minor	3	0.319914
50	D#-G-C	minor	3	0.165701
51	E-G#-C#	minor	3	0.240547
52	F-A-D	minor	3	0.157708
53	F#-A#-D#	minor	3	0.095246
54	G-B-E	minor	3	0.139431
55	G#-C-F	minor	3	0.282301

56	D-G-A#	minor	3	0.238416
57	D#-G#-B	minor	3	0.158455
58	E-A-C	minor	3	0.177239
59	F-A#-C#	minor	3	0.150824
60	F#-B-D	minor	3	0.261457
61	G-C-D#	minor	3	0.164032
62	G#-C#-E	minor	3	0.084516
MEDUIM COMPLEX (4 tones)				
155	G-A#-D-F	m7	4	0.35643
156	G#-B-D#-F#	m7	4	0.400771
157	A-C-E-G	m7	4	0.267497
158	A#-C#-F-G#	m7	4	0.257447
159	B-D-F#-A	m7	4	0.168
160	C-D#-G-A#	m7	4	0.238059
161	C#-E-G#-B	m7	4	0.337309
162	D-F-A-C	m7	4	0.240938
163	D#-F#-A#-C#	m7	4	2.621728
164	E-G-B-D	m7	4	2.523926
133	G-B-D-E	sixth	4	0.473332
134	G#-C-D#-F	sixth	4	0.404992
135	A-C#-E-F#	sixth	4	0.427638
136	A#-D-F-G	sixth	4	0.345792
137	B-D#-F#-G#	sixth	4	0.319823
138	C-E-G-A	sixth	4	0.774109
139	C#-F-G#-A#	sixth	4	1.0701
140	D-F#-A-B	sixth	4	0.651537
141	D#-G-A#-C	sixth	4	0.697279
142	E-G#-B-C#	sixth	4	0.479862
HIGHLY COMPLEX (5 or 6 tones)				
315	C#-D#-F-F#-G#-A#	hexatonic	6	1.961652
316	D-E-F#-G-A-B	hexatonic	6	1.950049
317	D#-F-G-G#-A#-C	hexatonic	6	2.587533
318	E-F#-G#-A-B-C#	hexatonic	6	1.493895
319	F-G-A-A#-C-D	hexatonic	6	1.481942
300	A-C#-E-G-B-D#	dom7plus11	6	3.114812
301	A#-D-F-G#-C-E	dom7plus11	6	2.235246
302	B-D#-F#-A-C#-F	dom7plus11	6	2.327629
303	C-E-G-A#-D-F#	dom7plus11	6	2.616966
304	C#-F-G#-B-D#-G	dom7plus11	6	1.923147
249	C#-E-G#-B-D#	m9	5	1.081435
201	A-C#-E-G#-B	maj9	5	1.194153
261	D-F#-A-C#-E	maj9	5	3.253967
262	D#-G-A#-D-F	maj9	5	3.20722

263	E-G#-B-D#-F#	maj9	5	2.014812
264	F-A-C-E-G	maj9	5	1.818317
276	G-G#-B-C#-E	neappenta	5	2.212012
279	A#-B-D-E-G	neappenta	5	1.706414
280	B-C-D#-F-G#	neappenta	5	1.504145
281	C-C#-E-F#-A	neappenta	5	1.13532

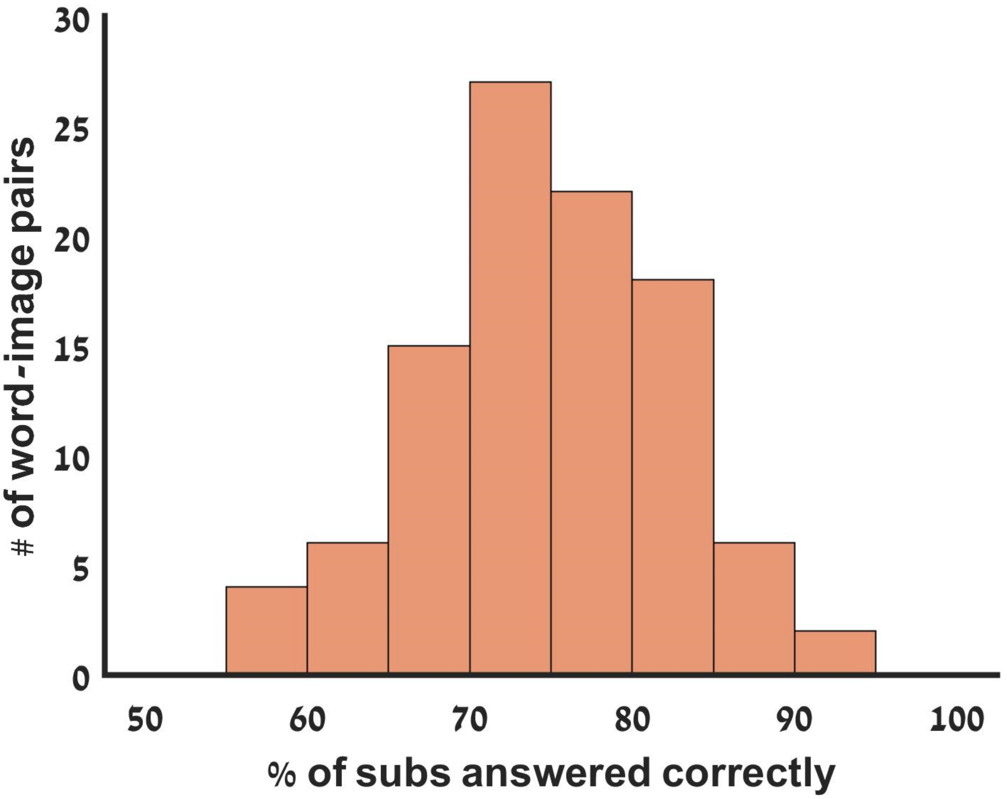

Figure S1. Distribution of the number of word-image pairs answered correctly (% of participants who answered correctly).
